# Sailing Away From the Sarcomere: The Mitral Valve in Hypertrophic Cardiomyopathy∗

**DOI:** 10.1016/j.jacadv.2023.100350

**Published:** 2023-05-26

**Authors:** Martin S. Maron

**Affiliations:** Division of Cardiology, Hypertrophic Cardiomyopathy Center, Lahey Hospital and Medical Center, Burlington, Massachusetts, USA

**Keywords:** heart failure, hypertrophic cardiomyopathy, mitral valve

**Figure:**
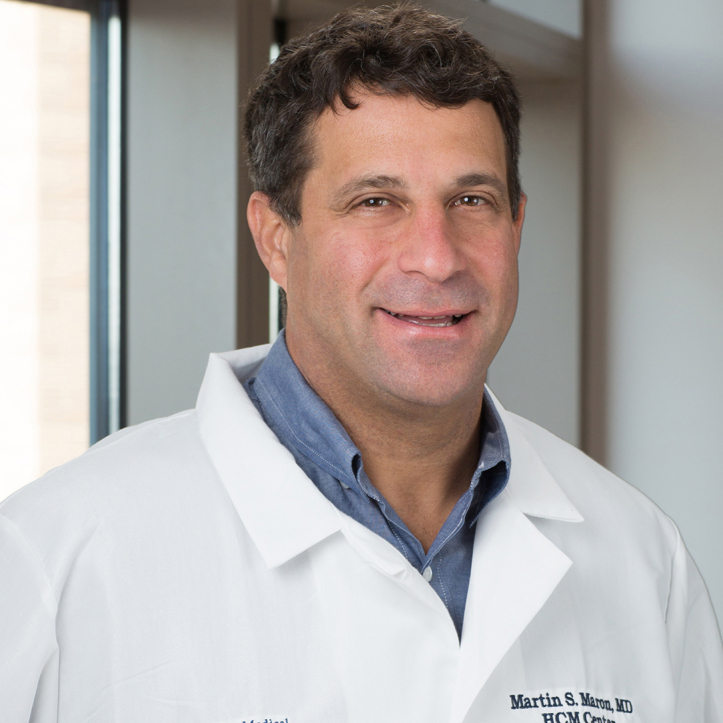

Since the initial descriptions of hypertrophic cardiomyopathy (HCM), left ventricular (LV) hypertrophy has been the *sine qua non* disease feature and the basis for clinical diagnosis.^1^ Over this time, a multitude of imaging and histologic studies have also identified a number of other morphologic abnormalities of the HCM heart outside of the LV myocardium.^2^^,^^3^ These have included abnormal intramural coronary arteries, interstitial and replacement fibrosis, displaced and hypertrophied papillary muscles and structurally abnormal mitral valve leaflets.^2^, ^3^, ^4^, ^5^, ^6^ The collection of these diverse features converge to produce a common HCM disease entity.^6^

Notably, the most visible of these disease features has been the mitral valve, given its fundamental role in dynamic outflow tract obstruction in HCM.^3^^,^^5^ Prior investigations have demonstrated that the mitral valve leaflets are elongated in a substantial proportion of HCM patients, often exceeding 2x the valve length of normal controls.^2^^,^^4^^,^^5^ The change in mitral valve structure is unrelated to age or other relevant demographic or disease variables, including magnitude of LV hypertrophy.^4^ These observations have suggested that morphologic abnormalities of the mitral valve in HCM represent an independent expression of this heterogeneous genetic heart disease, although the pathophysiologic mechanisms responsible for these changes to the valve has not been well defined.^4^, ^5^, ^6^

In this issue of the *JACC: Advances*, Troy et al^7^ provide a comprehensive characterization of the mitral valve in HCM, including detailed gross and histopathologic features. The authors accessed sections of 22 mitral valve leaflets excised during surgical myectomy and compared to controls without heart disease. Valve specimens were subjected to immunohistochemical stains with Hematoxylin and Eosin, trichrome, and elastic and staining for developmental dysregulation with epicardium-derived cell differentiation and paracrine signaling, adaptive endocardial-to-mesenchymal transition, interstitial proliferation, as well as cardiomyocytes.

This detailed inspection found that the histopathologic architecture of the mitral valve leaflets was markedly abnormal in HCM, characterized by a structurally disorganized and expanded amount of elastic fibers in the spongiosa layer and notable less dense and looser collagen in the fibrosa layer.^7^ The atrial and ventricular surfaces of the valve leaflet were also superimposed with collagen deposition, a consequence of long-standing mitral valve—ventricular septal contact. These changes to the inherent structure of the mitral valve are responsible for increasing leaflet length and flexibility and decreasing valve thickness, resulting in a myoxoid mitral valve that in many HCM patients appears like a loose sail.

Based on these cross-sectional observations, the authors propose that the mitral valve in obstructive HCM is engaged in a positive feedback loop in which the valve leaflets are exposed to chronic drag forces within the outflow tract area.^7^ As a consequence, over time, this insult promotes reactive proliferation resulting in further leaflet elongation. Ultimately, these structural changes of the mitral valve appear to potentiate dynamic outflow obstruction and alter valve coaptation increasing severity of mitral regurgitation. As the authors suggest, this may be one of the reasons why limiting symptom due to outflow tract obstruction begin for many patients in midlife (and for some not until advanced age) despite the fact that an HCM diagnosis has preceded symptoms often by many years.^8^

How do these unique observations translate to clinical management of HCM? Recently, there has been increasing attention on intervening earlier on outflow obstruction in less symptomatic patients as an approach to beneficially altering natural history.^9^ This principle was advanced in the 2020 AHA/ACC HCM guidelines in which select patients, such as those with increased left atrial size who have developed symptomatic paroxysmal atrial fibrillation, may be eligible for earlier surgical myectomy (at comprehensive HCM centers) before marked symptoms develop, in order to mitigate the chances for long-term sequelae.^8^

These data from Troy et al^7^ provide additional circumstantial evidence to expand this principle by suggesting that negative inotropic medical therapy or surgery earlier in the course of obstructive HCM patients could result in maintaining the structural integrity of the mitral valve (by decreasing exposure of the valve leaflet to outflow forces) and potentially mitigate future increases in outflow gradients and mitral regurgitation. However, additional evidence, particularly longitudinal studies that demonstrate changes to valve length over time, should be required before we can fully adapt earlier treatment intervention as a strategy that could mitigate adverse structural changes to the mitral valve and decrease risk for the development of progressive heart failure symptoms.

These data also tell us something potentially important about what may cause HCM. For example, for the feedback loop to begin, the mitral valve leaflets must be elongated initially.^4^^,^^5^^,^^7^ Therefore, there appears to be a primary process responsible for the morphologic changes to the valve, an observation also supported by prior imaging studies demonstrating that the mitral valve is also elongated in nonobstructive HCM patients, early in life and in gene positive family members without LV hypertrophy.^4^ Therefore, these data contribute to the totality of evidence suggesting that the increase in valve length may be a consequence of abnormal early cardiac development.^10^ However, in investigating this possibility, the authors found no difference in cells that impact heart development in HCM valves compared to controls. Cardiomyoctyes were also confirmed to be absent from the leaflets suggesting that sarcomere mutations are also unlikely to account for this morphologic expression of HCM.^7^ Therefore, a precise pathogenesis for primary elongation of the mitral valve leaflets in HCM patients still remains uncertain.

In conclusion, this study underscores the need to reconsider future investigative pathways beyond the sarcomere toward non-mutational mechanisms that may underlie HCM. The primary morphologic changes to the mitral valve represent a ‘call to action’ to fully accept that HCM as a complex heart disease in which all aspects cannot be explained by a single point mutation. Exploring other mechanisms that may well play an important role in disease expression, including modifier genes and environmental factors, will help us to understand how all of the components come together to form this common complex heart disease and in the process come closer to a cure.

## Funding support and author disclosures

Dr Maron is a Steering Committee Member for SEQUIA HCM Cytokinetics; and a consultant to Imbria pharmaceuticals and Edgewise.
